# The Role of TAM Family Receptors in Immune Cell Function: Implications for Cancer Therapy

**DOI:** 10.3390/cancers8100097

**Published:** 2016-10-21

**Authors:** Magdalena Paolino, Josef M. Penninger

**Affiliations:** IMBA, Institute of Molecular Biotechnology of the Austrian Academy of Sciences, Dr. Bohrgasse 3, A-1030 Vienna, Austria

**Keywords:** TAM receptors, Gas6, Protein S, cancer immunotherapy, NK cells, Cbl-b

## Abstract

The TAM receptor protein tyrosine kinases—Tyro3, Axl, and Mer—are essential regulators of immune homeostasis. Guided by their cognate ligands Growth arrest-specific gene 6 (Gas6) and Protein S (Pros1), these receptors ensure the resolution of inflammation by dampening the activation of innate cells as well as by restoring tissue function through promotion of tissue repair and clearance of apoptotic cells. Their central role as negative immune regulators is highlighted by the fact that deregulation of TAM signaling has been linked to the pathogenesis of autoimmune, inflammatory, and infectious diseases. Importantly, TAM receptors have also been associated with cancer development and progression. In a cancer setting, TAM receptors have a dual regulatory role, controlling the initiation and progression of tumor development and, at the same time, the associated anti-tumor responses of diverse immune cells. Thus, modulation of TAM receptors has emerged as a potential novel strategy for cancer treatment. In this review, we discuss our current understanding of how TAM receptors control immunity, with a particular focus on the regulation of anti-tumor responses and its implications for cancer immunotherapy.

## 1. Introduction

It has been more than two decades since Tyro3, Axl, and Mer were first identified and grouped as the subfamily of TAM receptor tyrosine kinases [1,2]. Since then, rigorous research has revealed crucial functions of these distinctive receptors in normal physiology and in a variety of diseases. TAM signaling has been implicated in controlling platelet aggregation and thrombus formation [3], erythropoiesis [4], and endothelial and vascular smooth-muscle homeostasis [5,6,7]. In addition, TAM-dependent pathways participate in spermatogenesis [8], functional maintenance of the retina and lactating mammary gland [9,10], bone physiology [11], atherosclerosis [12], nervous-system biology [13], and permeability of the blood–brain barrier [14]. Historically and currently, most research in the field focuses on the prominent role that TAM receptors play in two processes: cancer development and immune regulation.

TAM receptors are key pleiotropic inhibitors of the immune system [15]. Diverse immune cells, in humans and mice, express TAM components and are severely perturbed if ablated of TAM-dependent cellular pathways [16]. TAM signaling lies at the border of the innate and the adaptive immune systems, where it provides an indispensable inhibitory feedback mechanism responsible for safeguarding the shutdown of inflammation and for the promotion of tissue-repair processes after adaptive immune responses have been triggered [17]. Blocking TAM signaling has great bodily impact, causing severe defects in the clearance of apoptotic cells, widespread inflammation and over-activation of the immune system, and development of systemic autoimmunity [8,18].

Since their discovery, TAM receptors have been positively associated with cancer. Strong experimental evidence supports the consensus that TAM receptors, in a cell-autonomous manner, act as pro-oncogenes enhancing the growth, survival, migration, and epithelial-to-mesenchymal transition of tumor cells [19]. TAM receptors have also been implicated in boosting metastasis and resistance to chemotherapeutic agents [20,21,22,23], two of the most therapeutically challenging hallmarks of cancer. Notably, targeted inhibition of TAM signaling has proven to have robust anti-tumor efficacy in diverse experimental cancer settings. These results have encouraged a heated race in the development of novel and specific ways of inhibiting TAM signaling for use in cancer patients [24]. In fact, small-molecule kinase inhibitors, monoclonal antibodies, and soluble decoy TAM receptors are currently under development [25,26].

Surprisingly, few studies have been dedicated to directly exploring the role of TAM signaling in the context of tumor immunology. Nonetheless, these scarce studies are encouraging, as they expose a central role for TAM receptors and their ligands in the regulation of antitumor immunity [27,28,29]. Because current immunotherapy strategies concentrate on hindering key inhibitory checkpoints of the immune system in order to unleash robust antitumor responses [30], TAM signaling gains further relevance. TAM inhibition is expected not only to activate strong anti-tumor immune responses, but also to directly impede the tumorigenic, metastatic, and chemoresistance capabilities of the tumor cell. In this review, we summarize the current knowledge of the crucial functions of TAM receptors and their cognate ligands in the regulation of immune responses. We further discuss how alterations in TAM signaling impact anti-tumor immune responses and the expected benefits and possible adverse effects of TAM-based therapeutics for cancer treatment.

## 2. TAM Receptors and Ligands: A Brief Overview

**Figure 1:**
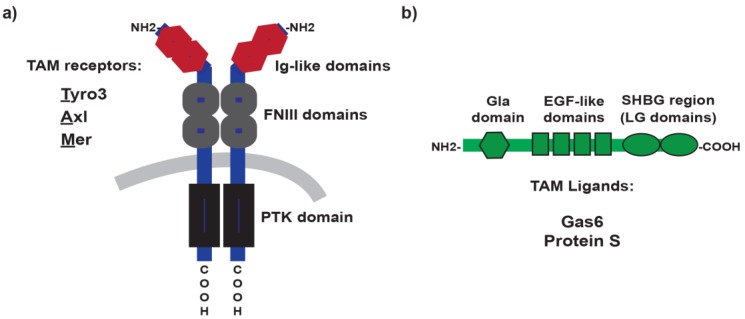
The structural domains of TAM receptors and ligands. Representation of the domain organization of the three TAM receptors: Tyro3, Axl and Mer at the plasma membrane of cells (**a**). TAM ligands: growth arrest-specific gene 6 (Gas6) and Protein S (Pros 1) are soluble circulating proteins (**b**). TAM receptors use their immunoglobulin-like (Ig-like) domains to bind the laminin G (LG) domains in the sex hormone binding globulin (SHBG) region of TAM ligands. PTK: Protein tyrosine kinase domain; FNIII: fibronectin type III domains; Gla: gamma-carboxyglutamic acid-rich domain; EGF-like: epidermal growth factor-like domain.

Tyro3, Axl, and Mer, compose the TAM subfamily of receptor tyrosine kinases (RTK). The classification is based on their distinctive domain structure and the unique KW(I/L)A(I/L)ES sequence in their catalytic domain [31,32,33]. The ecto-domain of these receptors exhibits two immunoglobulin Ig-like domains followed by two fibronectin type III domains. It is through the Ig-like domains that TAM receptors bind their ligands [25] (Figure 1). The hydrophobic transmembrane domain is of a single-pass type, and is followed by the intracellular portion that includes the tyrosine kinase domain, several autophosphorylation sites, and a conserved immunoreceptor tyrosine-based inhibitory motif-like (ITIM-like motif) [25]. The traditional agonist ligands for TAM receptors are the growth arrest-specific gene 6 (Gas6) and Protein S (Pros1) [34,35]. Both are soluble circulating proteins, which despite sharing 42% protein identity [36], exhibit distinctive specificities for TAM receptors [37]. Whereas Gas6 is able to activate all TAM receptors (Axl>Tyro3>>>Mer), Protein S is only capable of activating Tyro3 and Mer (Tyro3>Mer). In addition, three other TAM ligands have recently been described: Tubby, tubby-like protein 1 (Tulp-1) and Galectin-3 [38,39]. However, the physiological relevance of these ligands is still unclear.

In a basal state, TAM receptors are silent and display low kinase activity. Adhering to the dogma for RTK activation [2], initiation of the downstream phosphorylation cascades events occurs upon ligand binding induced dimerization of the receptor. Besides classical homodimerization, heterodimerization has also been suggested for TAM receptors [40]. Similarly, TAM ligands can form dimers [41]. Shedding of the extracellular domain upon receptor activation has been described, in particular for Axl and Mer [42,43], and is believed to be of physiological relevance, for instance, to acquire resistance to chemotherapy [23] or as decoy soluble receptors [44]. Similar to several proteins of the coagulation cascades, TAM ligands possess a gamma-carboxyglutamic acid-rich (Gla) domain [36]. The vitamin K-dependent γ-carboxylation of the glutamic acids residues in the Gla domain is required for TAM receptors to bind, via Ca2^+^, to the phospholipid phosphatidylserine (PtdSer) [45], exposed in the membranes of apoptotic cells, activated platelets, enveloped viruses, and activated T cells. Importantly, only γ-carboxylated ligands bound to PtdSer can significantly activate TAM signaling pathways [37,44]. Like many other oral anticoagulants, warfarin acts as a vitamin K antagonist to inhibit γ-carboxylation of the Gla domains in blood-clotting proteins [46]. By the same mechanism, warfarin can efficiently prevent γ-carboxylation of TAM ligands, and it is because of this property that warfarin is used as a TAM antagonist in research [44,47,48,49,50].

Cellular co-expression of two or even three receptors is exceptionally common in this family, and highly overlapping functions are often described [15]. Cells expressing TAM receptors frequently produce TAM ligands [15]. TAM receptors and ligands are predominantly expressed in myeloid cells, including macrophages [51,52], dendritic cells [17,53], monocytes [54], platelets [55], but also in NK [29] and NKT cells [56]. Although lymphocytes do not express TAM receptors [18], T lymphocytes express Pros1, but only upon cellular activation [57]. TAM components are additionally expressed in non-hematopoietic cells, such as neurons [13], osteoclasts [11], epithelial [9,10], and endothelial cells [7,58]. Despite their wide expression pattern and functional overlap, unusually for RTKs, TAM signaling appears dispensable for embryologic development, since TAM-deficient mice, including the triple knockouts, are viable [8].

## 3. TAM-Mediated Regulation of Immunity

It was not until the generation of gene-targeted mice that the relevance of TAM-mediated pathways in maintaining immune homeostasis became apparent. The first mouse line described was the Mer^KD^ catalytic dead line, where Mer kinase activity was inactivated [59]. These mice succumb to low doses of lipopolysaccharide (LPS), due to the immune hyper-activation and excessive production of pro-inflammatory cytokines, revealing for the first time an essential role for TAM kinase activation in dampening inflammation [59]. Later, total body knockout mice for each of the TAM receptors, as well as the combinatory double and triple TAM knockouts, were created [8]. Besides defects in retina, nervous, and reproductive organs [8], the prominent alterations associated with ablation of TAM receptors occurred within the immune system [18]. Notably, a gene dosage effect is evident, and most immunological phenotypes are visible or exacerbated only when Tyro3, Axl, and Mer are simultaneously ablated (TAM^−/−^ mice) [8,18].

**Figure 2:**
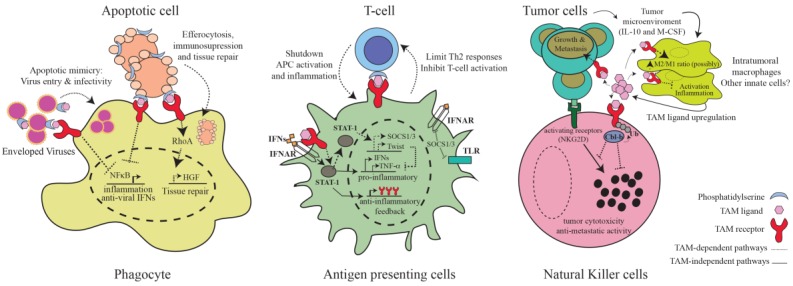
TAM-dependent immunity. Schematic representation of the major cellular functions of TAM receptors in the regulation of immunity. TAM-signaling functions as a pleiotropic inhibitory pathway in charge of resolving inflammation by dampening the activation of innate cells as well as restoring tissue function through promotion of tissue repair and clearance of apoptotic cells. Viruses can mimic apoptotic cell death to usurp TAM-dependent inhibitory pathways for their benefit. In a cancer setting, tumor cells induce TAM signaling to: (i) dampen NK-cell based anti-tumoral responses; (ii) reduce innate cells-mediated inflammation and possibly increase the ratio of M2-to-M1 intratumoral macrophages; and (iii) to directly promote its own growth and metastasis. A possible role for TAM signaling inhibiting Natural Killer T (NKT) cells has been proposed but is not presented here (see main text). A simplified view of the as yet identified underlying molecular mechanisms regulating these bioactivities is presented. Arrows indicate activating interactions. Flat-ended lines indicate inhibitory interactions. Ub: Ubiquitin; HGF: hepatocyte growth factor; IFNs: type-1 interferons; IFNAR: type-1 interferon receptor; NFkB: Nuclear Factor kB; RhoA: Ras homolog gene family member A; STAT-1: Signal transducer and activator of transcription 1; Th2: Lymphocyte T helper 2; APC: Antigen-presenting cell; TLR: Toll-like receptor; SOCS1/3: Suppressor of cytokine signaling 1/3; NKG2D: natural-killer group 2 member D; Cbl-b: Casitas B-lineage lymphoma-b; M1: classical macrophages; M2: alternative macrophages; IL-10: Interleukin-10; M-CSF: macrophage colony-stimulating factors; TNF-α: Tumor necrosis factor alpha.

At around one month of age, TAM^−/−^ mice start to display significantly enlarged spleens and lymph nodes, a common sign of lymphoid over-activation [8,18]. In these mutant mice, dendritic cells and macrophages are spontaneously activated and can secrete excessive inflammatory cytokines [18]. Transplant of TAM^−/−^ bone marrows into wild-type recipient animals recapitulated the hyper-proliferative phenotype of lymphoid cells, confining the defects to the leukocyte compartment of TAM-deficient mice [18]. By six months, these mice also exhibit generalized organ infiltration, high serological titers of autoantibodies, swollen joints, and IgG-induced glomerulonephritis [18], key clinical features of rheumatoid arthritis and systemic lupus autoimmune diseases. Mechanistically, it was postulated that, if TAM signaling is absent, the consequently excessive activation of innate cells results in aberrant activation of self-reactive lymphocytes [18]. This hypothesis pioneered the concept that TAM receptors control immune responses at the interphase of innate and adaptive immunity. Subsequent studies reinforced this notion, providing additional insight into the most prominent cellular and molecular mechanisms of TAM-dependent immunity. A summary of the major TAM-dependent immunological mechanisms is discussed below and is presented in Figure 2.

### 3.1. Inhibition of Inflammatory Pathways in Antigen-Presenting Cells

Inflammatory responses have to be limited in time and magnitude in order to prevent the development of detrimental autoimmune responses. The immune system employs diverse strategies to turn off inflammation, one crucial method being the activation of TAM signaling pathways. TAM receptors operate to prevent chronic activation of antigen-presenting cells (APCs) by dampening inflammatory pathways downstream of Toll-like receptors (TLR) and cytokine receptors [17,59]. Stimulation of APCs with Gas6 or Pros1 potently inhibits Nuclear Factor kB (NFκB) activation and cytokine production downstream of TLR3, TRL4, and TLR9 [17]. TAM-dependent pathways function as mechanisms of negative feedback in inflammation, and are only engaged after the inflammatory receptor has been activated. To assure this, TAM receptors themselves are under the control of TLR-induced signaling [17]. Upon activation of TLR receptors, a first torrent of pro-inflammatory cytokines is released, which then propagates inflammation by stimulating diverse cytokine receptors, including type I interferon receptors (IFNAR). It is at this moment when TAM receptor signaling is induced. IFNARs, via the Janus kinase/Signal transducers and activators of transcription (JAK/STAT) signaling pathway, upregulate TAM receptor expression in order to start a negative feedback loop that will inhibit type I interferon responses [17]. Induced TAM receptors then hijack the IFNAR signaling pathway to induce expression of the suppressor of cytokine signaling proteins (SOCS) [17]. TAM-induced Suppressor of cytokine signaling 1 (SOCS1) and SOCS3 E3 ubiquitin ligases in turn shutdown inflammation by ubiquitin-dependent inhibition of the proximal TLR and cytokine receptors signaling adaptors TNF receptor associated factor 6 (TRAF6) and TRAF3 [17]. It has been further described that type-I IFN-dependent induction of TAM receptors can also lead to the activation of Twist transcriptional repressors, which subsequently suppress the production of the potent inflammatory cytokine Tumor necrosis factor alpha (TNF-α) [60]. Although these mechanisms have been described for Axl, a similar involvement of other TAM receptors, in particular Mer, is highly suspected (Figure 2). This delayed negative feedback mechanism assures that the initiation of TAM inhibitory pathways only occurs at the end stages of the immune responses, allowing efficient inflammatory responses to occur while preventing chronic inflammation and concomitant tissue damage.

### 3.2. Phagocytosis of Apoptotic Cells

The second prominent immunoregulatory function of TAM receptors involves the regulation of phagocyte-dependent clearance of apoptotic cells [61], a process also known as efferocytosis [62]. The first TAM-dependent defects in homeostatic phagocytosis were observed outside the immune system. Adult mice expressing the kinase-dead versions of Mer are unable to clear the apoptotic debris periodically generated in the retina and testis, which results in blindness and sterility, respectively [8]. A recent study reports similar TAM-related defects in microglial phagocytosis of apoptotic cells during adult neurogenesis and brain damage [63]. Likewise, innate cells specialized in phagocytosis, namely macrophages and dendritic cells, show profound defects if TAM signaling is disrupted. Although Mer is the most implicated receptor for this phagocytic role [64], each of the three TAM receptors appears to contribute [40]. Notably, the phagocytic defect is specific for apoptotic cells, as the absence of TAM signaling does not preclude phagocytosis of bacteria or synthetic particles [64]. The high selectivity for apoptotic phagocytosis relies on the mechanism of TAM activation. As mentioned previously, Gas6 and Pros1 ligands only strongly activate TAM receptors when bound to PtdSer residues via their Gla domains [37]. Importantly, cells undergoing apoptosis promote their own TAM-mediated clearance by exposing PtdSer in their membranes, as a potent “eat me” signal that differentiates them from healthy cells or necrotic cells [65]. Following a tripartite model, TAM ligands function to bridge the interaction and activation of the phagocyte with the apoptotic cells [37].

Numerous tissues are under constant remodeling and regeneration. During these periodic processes, clearance of apoptotic cells needs to occur in a tolerogenic manner to maintain tissue functionality and homeostasis [66]. Importantly, in addition to mediating Vav guanidine nucleotide exchange factor 1/Ras homology family member A/Ras related botulinum toxin substrate 1 (Vav1/RhoA/Rac1)-dependent actin-cytoskeleton rearrangements required for phagocytosis [67,68], TAM receptors are also responsible for mediating the immunosuppressive effects of apoptosis (Figure 2). In experimental models, deregulation of Mer-dependent apoptotic pathways prevents the generation of tolerogenic dendritic cells upon cellular apoptosis, generating instead dendritic cells with enhanced T cell stimulatory capacity [53]. These defects have been associated with exacerbated susceptibility to autoimmunity [53]. Axl^−/−^Mer^−/−^ double mutant mice are also unable to clear neutrophils in the large intestine after dextran sulfate sodium (DSS) stimulation, resulting in enhanced colitis [69]. Although the molecular signaling details are yet to be revealed and compared to those involved in TAM-mediated regulation of inflammation, Mer studies suggest that TAM receptors can translate apoptotic phagocytosis into inhibition of NFκB activation and secretion of pro-inflammatory cytokines [70]. Additionally, cholesterol-dependent activation of Liver X receptor (LXR) transcription factors has been shown to upregulate the expression of Mer in macrophages as a positive feedback mechanism promoting engulfment of apoptotic cells and immunosuppression [71]. In addition, efferocytosis triggers regenerative responses to repair damaged tissues. Mer receptor and Gas6, but not Axl and Tyro3, appear to contribute the most by guiding, via RhoA activation, the upregulation of hepatocyte growth factor, a key effector of tissue reparation [72]. Thus, TAM receptors are responsible for creating an anti-inflammatory and reparative setting around apoptotic sites that prevents inflammatory responses towards self-derived antigens and restores tissue homeostasis.

### 3.3. TAM Receptors as Integrators of Innate and Adaptive Immunity

TAM receptors regulate the bilateral communication between dendritic and lymphoid T cells in different immunostimulatory conditions. Once activated, adaptive immune cells must communicate back with innate cells to avoid uncontrolled and chronic activation of the immune system. Very early studies noticed that Pros1 expression was upregulated in T cells upon activation [73], and when bound to the surface of T cells, Pros1 could inhibit T cell proliferation [74]. At that time, TAM signaling had not yet been implicated in immunity, and so the results were deduced in the context of Pros1 involvement in coagulation. Almost 20 years later, these results were confirmed and their functionality reinterpreted. Pros1 is expressed on the surface of T cells upon in vitro or in vivo activation, but not in resting T cells [57]. Although Pros1 could be secreted to act independently, the fact that moderate levels of PtdSer are simultaneously exposed at the plasma membrane of activated T cells strongly suggests that Pros1 acts locally to mediate the direct interaction of T cells and dendritic cells [57]. Indeed, evidence shows that Pros1 functions in T cells to signal back to TAM receptors expressed in antigen presenting cells, i.e., Axl and Mer, as a negative inhibitory feedback mechanism that controls the scale of antigen-specific immune activation (Figure 2). T-cell-specific ablation of Pros1 leads to a significant increase in the percentage of activated dendritic cells producing TNF-α and Interleukin-6 (IL-6) inflammatory cytokines, which, in colitis mouse models, conducts to pathological inflammation [57]. Likewise, Pros1 acts similarly in human T cells to repress activation of dendritic cells [57].

TAM receptors can also communicate back to T cells to control the magnitude and quality of adaptive immune responses (Figure 2). Neutralization of Mer receptors in human dendritic cells results in enhanced T cell proliferative cytokine responses, showing that TAM signaling can directly inhibit T cell activation [75]. Recently, a central role for Tyro3 receptors in limiting type 2 immunity against helminthes and allergens has been revealed, both for humans and for mice [76]. Interestingly, Tyro3 is only involved in lymphocyte T helper 2 (Th2) responses, without affecting the Th1 counterparts [76]. This specificity is assured by the fact that IL-4, the master cytokine in Th2 commitment, maintains the expression of the Tyro3 and Pros1, the major TAM signaling components involved in TAM-dependent Th2 regulation [73,76]. Thus, TAM signaling functions as a bi-directional integrator of the innate and adaptive immune systems, capable of adapting the magnitude and specificity of immune responses.

### 3.4. NK and NKT Cells

TAM signaling also is crucially involved in the development of natural killer (NK) cells [77]. In mouse bone marrow, stromal cells produce Gas6 and Pros1 to support the end-stage differentiation of NK cells via TAM receptor signaling [78]. All three TAM receptors are expressed on the surface of developing NK cells, and when ablated, NK cells fail to acquire the balanced distribution of activating and inhibitory receptors required for proper functionality [78]. NK cells from TAM^−/−^ mice move to the periphery in an immature state, precluding them from mounting cytotoxic and cytokine responses [78]. In vitro studies suggest this function is conserved in human NK cells, where blockage of the Axl/Gas6 axis resulted in similar NK cell developmental defects [79]. Similar to the regulation of IFNAR-dependent inflammatory responses, TAM receptors interact with cytokine receptors for the regulation of NK cell development, in this case Interleukin-15 receptors [78]. Apart from regulating NK cell development, we have recently demonstrated that TAM receptors, via Casitas B-lineage lymphoma-b (Cbl-b)-mediated ubiquitylation, constitute a novel negative pathway in NK cell activation, controlling NK cell proliferation, IFN-γ production, and cytotoxic responses [29] (Figure 2). Importantly, this pathway has great implications for tumor immunotherapy, as will be discussed in-depth later.

An additional role for TAM signaling in Natural Killer T (NKT) cells has been implied [56]. NKT cells express Mer receptors on the cell surface, and if inactivated, in vivo activation of NKT cells with α-galactosylceramide is enhanced; Mer^KD^ NKT cells are hyperactivated and secrete higher levels of pro-inflammatory cytokines [56]. This defect is cell intrinsic, and does not appear to arise from a developmental deficiency, as similar numbers of NKT populations can be detected between wild-type and triple TAM knockout mice [78]. Whether the described TAM-mediated regulation of NKT has physiological relevance, and if so, in which settings, awaits experimentation.

### 3.5. Virus Entry and Infectivity

Intriguingly, viruses have evolved mechanisms to usurp TAM-dependent efferocytosis pathways for their own benefit, further emphasizing the central immunosuppressive role of TAM receptors. Enveloped viruses can expose PtdSer residues on the external leaflets of their envelopes in order to mimic an apoptotic cell, and by doing so, engage, via Pros1/Gas6, TAM receptors on the surface of antigen presenting cells [80] (Figure 2). Additionally, it has been reported that a non-enveloped virus, Simian vacuolating virus 40 (SV40), can directly bind TAM receptors by mimicking the structural properties of TAM ligands [81]. These apoptotic and ligand mimicry strategies allow viruses not only to infect innate immune cells but also to activate TAM signaling in order to attenuate type I interferon antiviral responses [82]. Indeed, viral infections have been shown to trigger the upregulation of TAM receptors [83]. It is also known that whereas TAM kinase activity is not required for virus entry, it is indispensable for viral replication [84]. Apoptotic mimicry and appropriation of TAM signaling has been described in the infectivity of a variety of relevant pathogenic human enveloped viruses, such as Ebola [85], Dengue and West Nile virus [84], and most recently, Zika virus [86]. Notably, whereas in vitro and at a cellular level, blockage of TAM receptors can efficiently impair virus infectivity and replication in dendritic cells [84,85,87], the net antiviral in vivo consequences of TAM inhibition seem to contradict the predicted therapeutic benefits [88]. The excessive type I interferon secretion observed in the absence of TAM signaling has been shown instead to increase susceptibility to influenza and West Nile virus infections, as the inflammatory environment resulting from TAM signaling disruption precluded dendritic cells from priming adequate antiviral T cell responses [88]. Additional studies are needed to better determine the overall benefits of targeted TAM inhibition for treatment or prevention of specific viral infections.

## 4. Functional Diversification for TAM Receptors

Triple and double TAM knockout mice reveal an apparent functional redundancy between Tyro3, Axl, and Mer in immune regulation [8,18]. However, recent efforts have started to illuminate degrees of functional dedication for TAM receptors and their ligands [37,44,89]. Firstly, although receptor co-expression can be detected in several immune cells in diverse tissues, a preferred differential expression is observed; whereas Axl shows a comparatively higher expression on dendritic cells, Mer is the predominant receptor in macrophages [37]. Of note, tissue location can alter these patterns [15,37]. Probably because of this distinctive expression profile, initial studies suggested a coherent predominant role for Axl and Mer in distinctive TAM-mediated events in these cells [17,64].

Interestingly, it has now been revealed that it is the immunological environment that alters the expression patterns of TAM receptors, and in turn, their immunological bioactivities, regardless of the cell type [37]. Tolerogenic agents instruct the immune system to use Mer receptors for the phagocytosis of apoptotic cells and the subsequent mediation of tissue-repair responses. Immunosuppressive glucocorticoids, such as dexamethasone, greatly induce the expression of Mer while simultaneously repressing Axl transcription in both dendritic cells and macrophages [37]. Hydrocortisone, aldosterone, and progesterone also can promote Mer expression [37]. In the absence of Mer, Pros1-induced phagocytosis of apoptotic cells by glucocorticoids is impaired [90]. On the contrary, inflammatory agonists such as Interferon alpha (IFN-α) or polyinosinic:polycytidylic acid (poly (I:C)) strongly upregulate the expression of Axl while reducing Mer levels in dendritic cells and macrophages [37,83]. In this scenario, Axl takes over the phagocytic role of engulfing apoptotic cells to engage the TAM-dependent inhibitory pathways required to resolve inflammation. In summary, whereas Mer, activated by Gas6 or Pros1, is predominantly responsible for homeostatic phagocytosis in immunosuppressive environments, Axl, activated by Gas6 but not Pros1, preferentially regulates phagocytosis and negative feedback pathways under inflammatory conditions. Importantly, evolutionary conservation of these immunoregulatory mechanisms has been demonstrated in human cells [83,90].

Apart from the recently revealed function of Tyro3 in controlling Th2 responses [76], little else is known about the physiological roles of Tyro3 in immunity. Basal expression of Tyro3 in dendritic cells is low, and it is even undetectable in macrophages [37]. Inflammatory stimuli decrease Tyro3 expression but only minimally, and no significant upregulation was observed with tolerogenic agonists [37]. Additionally, absence of Tyro3 does not perturb Mer- or Axl-dependent phagocytosis of apoptotic cells after dexamethasone and poly(I:C) stimulation [37]. Thus, Tyro3 functions in the regulation of apoptotic cell clearance and inhibitory inflammatory pathway in macrophages and dendritic cells will require additional rigorous investigation. Interestingly, TAM receptors have been shown to regulate immune responses by acting on non-immune cells [91,92]. All three TAM receptors are expressed in Sertoli cells [8,93] where they maintain testicular immune homeostasis by controlling the phagocytosis of apoptotic spermatogenic cells [94], as well as by negatively regulating the production of inflammatory cytokines downstream TLR3 receptors [91]. It would be interesting to expand the study of this novel TAM-dependent immunoregulatory mechanism to other epithelial tissues.

## 5. TAM Signaling in Autoimmunity

In humans, there is vast evidence showing an association between autoimmunity and abnormalities in TAM signaling components. Polymorphisms in the Mer gene are associated with systemic lupus erythematosus and multiple sclerosis [95,96]. Additional relations exist between single nucleotide polymorphisms (SNPs) in the Pros1 and Gas6 genes and patients with Behcet’s uveitis or type 2 diabetes [97,98]. Aberrant expression of TAM members also has been reported in autoimmune disorders. For instance, reduced expression of TAM receptors in circulating immune cells as well as low plasma concentrations of TAM ligands are evident in lupus, Behcet’s disease, rheumatoid arthritis, inflammatory bowel disease, and psoriasis patients [58,99,100,101,102,103]. Interestingly, elevated concentrations of circulating soluble TAM receptors have also been detected in patients with lupus, Sjogren’s syndrome, rheumatoid arthritis, and Behcet’s disease [95,104,105,106,107], which suggest that TAM receptor shedding could have an important role in the pathogenesis of autoimmunity.

In mice, as previously mentioned, triple TAM deficiency results in spontaneously develop systemic autoimmune responses that resemble lupus and rheumatoid arthritis [18]. Recently, an autoimmune hepatitis phenotype has been further described in these mice [108]. Of note, despite spontaneously developing autoimmunity, TAM-deficient mice have a normal life expectancy [8]. Lastly, also observed in mice, TAM deficiency enhances susceptibility to autoimmune experimental models of type 2 diabetes, experimental autoimmune encephalomyelitis, and rheumatoid arthritis [53,109,110]. Interestingly, administration of Gas6 or Pros1 resulted in anti-inflammatory therapeutic benefits in collagen-induced arthritis [111], as well as reduced in vitro demyelination in a cuprizone-induced model for multiple sclerosis [112]. This suggests that reconstitution of TAM signaling pathways could be of use for the treatment of autoimmune disorders. However, this hypothesis has yet to be tested in clinical trials.

Keeping in mind that animal studies were performed in total body knockout mice, an exclusive causal role for TAM-defective immune response in the pathogenesis of autoimmunity cannot be firmly concluded yet. However, most evidence points in this direction. For instance, transplanting wild-type leukocytes can reverse the spontaneous liver damage observed in TAM^−/−^ mice [108]. In a T cell receptor (TCR)-transgenic diabetes model, where autoimmunity is triggered by pancreatic beta-islets apoptosis, wild-type transgenic T cells become highly activated and inflammatory when transplanted into Mer^KD^ mutant mice [53]. If dendritic cells are depleted, the hyperactivation of the autoreactive T cells significantly diminishes in this model [53]. Furthermore, studies performed in two independent Mer mutant mouse lines have linked the inefficient clearance of apoptotic cells with increased production of autoantibodies and enhanced B and T cell activation [113,114]. Ineffective clearance of myelin debris in the absence of Axl has been associated with enhanced autoimmune susceptibility in experimental autoimmune encephalomyelitis (EAE) models of multiple sclerosis [110]. Accumulation of apoptotic cells and debris can cause tissue toxicity, as shown in the testes and retinas of TAM-deficient mice [8]. Moreover, prolonged accumulation of apoptotic cells is predicted to aberrantly over-expose self-antigens and can further result in secondary necrosis [115]. It is commonly believed that, if presented by APCs in an inflammatory milieu, self-antigens can break down peripheral immunotolerance and activate self-reactive lymphoid cells [115]. Finally, buildup of apoptotic cells has been associated with human autoimmune disorders, particularly lupus [116]. Taken together, evidence strongly suggests that the autoimmune phenotype of TAM^−/−^ mice arises from the detrimental combination of accumulation of apoptotic debris and unresolved chronic inflammation. Additional experimentation is needed to determine if other cellular or molecular mechanisms participate.

## 6. TAMing Anti-Tumor Immunity

**Figure 3:**
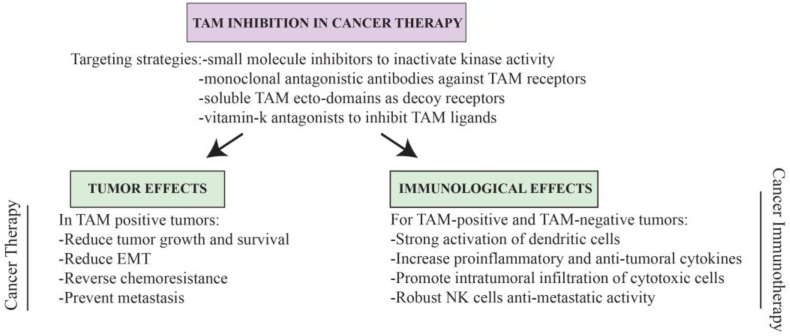
TAM signaling for cancer therapy. Different strategies for targeted TAM inactivation and the expected anti-cancer benefits of such therapies by directly affecting the tumor, or indirectly, via activation of the immune system. Possible side effects are discussed in the text. EMT: Epithelial–mesenchymal transition; NK: Natural-killer.

Vast literature supports the cell autonomous carcinogenic role of TAM signaling in tumor cells, where TAM antagonists are commonly used as anti-tumor strategies [19,117]. These extensive studies have established a consensus that TAM signaling has a crucial pro-oncogenic role in the initiation and progression of human cancers [49,118,119,120,121]. This hypothesis has been confirmed in a variety of human cancer cell lines and patient’s primary tumors including esophageal, melanoma, myeloid leukemia, prostate, multiple myeloma, intestinal, pancreatic, hepatic, ovarian, lung, thyroid, and brain tumors, among possibly many others [19]. In most of these cases, aberrant overexpression of TAM receptors is observed; mutations in TAM receptors are an exception [19]. Mechanistically, TAM receptor signaling supports several key cellular events in the tumor, from cell growth and survival, to metastasis, epithelial-mesenchymal transition (EMT), and resistance to chemotherapy [20,22,23,24,122,123,124,125]. Importantly, experimental evidence demonstrates that TAM antagonism can efficiently revert these processes [22,126,127,128,129,130], underscoring the potential benefits of TAM inhibition for cancer therapy (Figure 3). Together, these consistent results have encouraged the development of novel and specific ways of inhibiting TAM signaling in tumor cells for clinical use [24,131,132]. In fact, the first clinical trial with an anti-Axl specific small molecule inhibitor for treatment of acute myeloid leukemia and non-small cell lung cancer is currently underway [133], and several others newly synthetized inhibitors specific for TAM receptors are being tested at a preclinical stage [134,135].

It is somehow surprising that, given the pleiotropic inhibitory role of TAM receptors within the immune system, there has been comparatively less research exploring the immunological consequences of TAM inhibition in the context of cancer. Most in vivo studies exploring the cell autonomous pro-oncogenic role of TAM receptors employ human cancer cells in immunodeficient mice [49,123,126,127,136], therefore preventing the elucidation of the immune system’s contribution. The first indication that TAM signaling affects anti-tumor immunity came from genetic studies of Gas6-deficient mice, where absence of Gas6 in hematopoietic cells markedly impaired tumor proliferation and metastasis in diverse ectopic and orthotopic tumor models, including pancreatic, lymphoma, colon, breast, and melanoma tumors [27]. Mechanistically, it was revealed that the tumor is able to instruct intratumoral macrophages to overexpress and secrete Gas6 by releasing Interleukin-10 (IL-10) and Macrophage colony-stimulating factor (M-CSF) into the microenvironment [27] (Figure 2). Macrophage-derived Gas6 allegedly then serves to feed TAM-dependent pathways in the tumor cells [27], as an additional mechanism by which tumors alter the surrounding stroma for their own benefit. Likewise, stromal cells in the bone marrow upregulate Gas6 when multiple myeloma or metastatic prostate tumor cells are present, positively impacting the malignancy of these tumor cells [21,49].

Although certainly relevant, these reports focused on the effects of TAM signaling on the tumors and did not explore alteration in tumor immunity. Additionally, the experimental approach of modulating Gas6 and using tumors that express TAM receptors complicates interpretations; Gas6-deficiency can alter both the tumors and immune responses. Another study, employing TAM receptor deficient mice and TAM-negative tumor cells, was able to reveal for the first time that TAM signaling directly affects anti-tumor immunity [28]. Absence of Mer receptors markedly augmented serological levels of inflammatory cytokines and resulted in higher accumulation of cytotoxic cells in the tumor microenvironment [28] (Figure 3). As a result, Mer-deficient mice were able to control the growth and, importantly, metastases of breast, colon, and melanoma tumors [28]. Bone marrow transplants confirmed the sufficient anti-tumoral role of Mer^−/−^ leukocytes, whereas antibody depletion of CD8^+^ cells revealed the active involvement of cytotoxic T lymphocytes in tumor rejection [28]. That study also found that transcriptional upregulation of Gas6 could be detected in the microenvironment of melanoma and breast cancer tumors. Although in-depth mechanistic data were limited for this study, the authors suggest that the production of Gas6 by the tumor microenvironment promotes tumor progression by inhibiting, via activation of TAM signaling, the inflammatory innate responses required for activation of anti-tumor cytotoxicity.

We have recently revealed an additional and unforeseen mechanism of TAM-mediated regulation of NK cells that drastically affects anti-tumor responses [29]. Our initial in vitro studies revealed that mature NK cells express each of the TAM receptors, and upon activation with Gas6, proliferation of and IFN-γ production by activated NK cells is suppressed. Mechanistically, we identify that the E3 ubiquitin ligase Cbl-b is the crucial downstream mediator of TAM signaling in NK cells. Upon TAM activation, Cbl-b is recruited to TAM receptors at the plasma surface, where it mediates their ubiquitylation and concomitant downregulation from the cell surface. In the absence of Cbl-b, TAM receptor signaling is severely impaired, resulting in robust cytotoxic and inflammatory NK cell responses [29]. Thereby, we established that TAM receptors/Cbl-b constitutes a novel and relevant inhibitory pathway for NK cell activation. These data are consistent not only with the pleiotropic role of TAM signaling in inhibiting diverse innate cells but with the presence of an inhibitory immunomodulatory ITIM-like motif in the cytoplasmatic tails of TAM receptors [18], a domain peculiarity also shared by many NK cell inhibitory receptors [137].

From this finding we formulate the idea that TAM kinase inhibition could be used for NK-cell-based anti-metastatic therapy in vivo, and developed a novel small molecule inhibitor, termed LDC1267, highly selective (at low nanomolarity) for the three TAM receptors [29]. Oral administration of this inhibitor indeed derepressed NK cell activation and conferred strong anti-tumor NK cell activity against RMA-Rae1 tumor cells and metastatic B16F10 melanomas. Furthermore, an adoptive transfer experiment with LDC1267-treated NK cells into melanoma bearing mice was equally effective, confirming that the mere inhibition of TAM-dependent pathways in NK cells is sufficient to reduce tumor metastasis. Strikingly, TAM blockage was efficient even if drug treatment started after tumor metastasis; administration of LDC1267, including orally, resulted in significant reduction in tumor growth and metastasis in both melanoma as well as orthotopic metastatic breast cancer models [29]. In all tumor models, we confirmed LDC1267 has no apparent effect if NK cells are ablated. TAM signaling in the absence of Cbl-b is equally impaired as if TAM receptors are blocked with the kinase small molecule inhibitor [29], confirming the crucial role of Cbl-b downstream TAM signaling in NK cells. Thus, in different model systems and using different routes of administration, in vivo therapy with the selective TAM inhibitor LDC1267 consistently reduced metastases dependent on NK cells (Figure 3).

Our findings also provide a surprising molecular explanation of an old puzzle in cancer biology. More than 50 years ago, it was found in mouse and rat cancer models that the widely used anticoagulant warfarin has anti-metastatic properties that are dependent on its vitamin K antagonistic functions [138,139,140]. Since then, the underlying mechanisms had remained hypothetical [141,142]. Clinical studies in humans have also revealed a positive association between the use of warfarin and reduced cancer incidence as well as increased patient survival. However, these results have only been consistent for prostate cancer [143,144,145,146] and small-cell lung carcinoma [147,148,149,150]. Additionally, these studies have not determined the impact of warfarin on tumor growth and metastasis, and provided no insight into the mechanisms mediating the anti-cancer benefits of vitamin K antagonist therapy.

In our experiments, low doses of warfarin, which block TAM activation without affecting blood coagulation [48,151], indeed markedly reduced lung metastases in wild-type mice but had no further effect in Cbl-b mutant mice [29]. Upon NK cell depletion, warfarin anti-metastatic activities disappear [29]. Thus we proposed a first plausible model for warfarin’s anti-metastatic activity, in which warfarin mediates rejection of metastatic tumors by blocking the activation of TAM/Cbl-b inhibitory circuits in NK cells [29]. One year later, another group showed that low doses of warfarin efficiently block Axl signaling in tumor cells, preventing the progression and metastasis of pancreatic cancer by reversing EMT transition, tumor migration, chemoresistance, and intratumoral microvessel density [47]. These interesting findings call for future experimentation to revise the use of vitamin K antagonists as anti-TAM therapeutics.

Although these results are encouraging for their potential utility in cancer immunotherapy, it is important to note that not all evidence indicates that blocking TAM receptor signaling in the immune system would be beneficial in a cancer context. Although in vitro blockage of TAM signaling has potent anti-tumorigenic capacities towards numerous colorectal cancer cell lines [129], and ectopically inoculated colon cancer cells are rejected in TAM-deficient mice [27,28], in vivo ablation of TAM-dependent pathways in colitis-driven colorectal cancer appears to have pro-tumorigenic net effects [152]. Two independent groups observed that the marked susceptibility to experimentally induced colitis observed in Gas6^−/−^ as well as in double Axl^−/−^ Mer^−/−^ mutant mice correlates with increased incidence of colon cancer in genetically (APC^min^) as well as chemically induced colon cancer (AOM-DSS), resulting in larger tumors and reduced overall survival [69,153]. Moreover, intratumoral expression of Gas6 has been associated with positive prognosis in colorectal cancer patients [153]. Additionally, murine T cells lacking Pros1 became highly colitogenic when transplanted into Rag^−/−^ mice [57], and Pros1 deficiency has been detected in patients with ulcerative colitis or Crohn’s disease [102,103]. This hints that blocking Pros1 in vivo could also result in colitis-induced tumor progression.

Although more detailed cellular and molecular insights into the TAM-dependent anti-tumor mechanisms in this inflammatory setting are needed before a definite interpretation can be formulated, these data are consistent with the fact that chronic inflammation has long been established as a promoting factor in cancer [154]. Importantly, these studies strongly suggest that TAM blockage can be counterproductive in generating an excessively aggravated inflammatory response that promotes tumor progression, at least for inflammatory-driven cancer. These seemingly contradictory results are reminiscent of those observed in airway infection models, where TAM inhibition also had a counterintuitive net effect of increasing viral susceptibility due to over-exaggerated production of anti-viral cytokines [83]. Whether the constant exposure to microbiota and the different setup of the immune system in mucosal interfaces are responsible for this discrepancy still needs to be clarified. Nevertheless, these results call to consider a more controlled modulation of TAM signaling pathways in order to avoid excessive inflammation.

## 7. Conclusions and Perspectives for TAM-Based Cancer Therapy

Modulation of the immune system for the treatment of primary and metastatic tumors in cancer patients has been a goal for many decades [155]. Several modalities of cancer immunotherapy exist, varying from those attempting to modulate the immunogenicity of the tumor to those aiming at preventing immunotolerance and/or enhancing the cytotoxic responses of effector immune cells [156]. However, until recently the success of most cancer immunotherapies have been limited [157]. Fortunately, we are experiencing a new era for cancer immunotherapy, where targeted inactivation of key inhibitory immune pathways has succeeded in the robust stimulation of a patient’s own immune system to attack tumor cells [30,158,159]. In this context, targeted TAM inhibition gains prime relevance for cancer therapy.

Numerous observations make us believe that therapeutic anti-cancer regimens could benefit from the modulation of TAM receptor signaling and, in particular, its kinase activity. Firstly, TAM receptors have a pleiotropic role inhibiting diverse immune cells, including macrophages, dendritic cells, NK cells, NKT cells, and, indirectly, T cells [17,18,29,40,53,56,57], all of which are crucially implicated in anti-tumoral immunity. Thus TAM signaling inactivation can promote anti-tumor immunity at multiple levels, affecting diverse molecular and cellular mechanisms. Importantly, most TAM-dependent immune functions are also conserved in humans, where absence of TAM signaling pathways leads to strikingly similar defects in dendritic cells, macrophages, and NK cells [57,79,90,160,161]. Secondly, with the possible exception of inflammatory-driven cancer, experimental evidence has demonstrated that TAM signaling has a general role in tumor immunity. To date, and including our own results, different strategies of TAM inactivation have been tested in more than eight independent mouse tumor models giving highly consistent results: absence of TAM components or inactivation of TAM receptor kinase activity induces a spontaneous immune-mediated rejection of a variety of tumors including ectopic and orthotopic transplantable tumors, metastatic and genetically-driven spontaneous tumors [27,28,29]. Additionally, absence of TAM signaling can efficiently control anti-metastatic immune responses [28,29,47], an attribute extremely relevant for treating this devastating ability of malignant tumors. Thirdly, the structural and biochemical characteristics of TAM receptors and ligands offer multiple possible targeting strategies, including monoclonal antibodies to block the receptors at the cell surface or induce their internalization, specific small molecules to inactivate the kinase domain, soluble TAM ectodomains as decoy receptors, or even possibly repurposing vitamin K antagonists, such as warfarin, to inhibit TAM ligands. Importantly, we have shown that selective TAM inhibition with small molecules can efficiently mediate tumor rejection even when administrated orally [29], constituting another advantage for this type of therapy. Finally, we particularly want to highlight the unique potential dual benefit of targeting TAM signaling pathways in cancer therapy, where not only are anti-tumor responses expected to be unleashed, but importantly, as evidence unequivocally demonstrates, TAM-positive tumors, which are particularly common in humans, will also be directly precluded from growth, epithelial-to-mesenchymal transition, metastasis and chemoresistance (Figure 3).

Nevertheless, as demonstrated in colitis-induced cancer models, fine-tuning the duration and amplitude of inflammatory response in the absence of TAM signaling would be preferred in a TAM-based therapeutic setting to avoid chronic inflammation possibly driving tumorigenesis as well as autoimmunity. To this end, further mechanistic insights into TAM-mediated regulation of anti-tumor immune responses are needed. TAM research historically has been impeded by the absence of strategies to restrict TAM inhibition to certain cells or tissues. The diverse roles for TAM receptors and ligands inside and outside of the immune system, including within the tumor itself, complicate the interpretation of most studies in the field, where mainly systemic TAM ablation was utilized [16,19]. Recently, Axl and Pros1 floxed conditional mice have been generated [88,162], and similar efforts for the remaining TAM components will be equally helpful. It would also be necessary to better understand the biofunctional diversity of TAM receptors and ligands and to discriminate the effects of transient vs. constitutive inactivation of TAM signaling. A better understanding of the extent of the inflammatory responses and the TAM-dependent components, and of the cellular and molecular mechanisms involved in particular tumor settings would be key to help tailor TAM-targeted therapy in diverse cancer settings.

Importantly, evidence suggests that putative TAM-based cancer immunotherapies could have tolerable side effects. Although systemic autoimmunity develops in complete absence of TAM signaling (triple knockout mice), its consequences are well tolerated and do not reduce life expectancy [8]. Furthermore, only genetic deletion of all three TAM receptors results in overt autoimmunity [18], so partial inactivation of TAM signaling, as can be expected from kinase inhibitors or monoclonal antibodies, or selective inactivation of the identified key TAM components, is likely to provide a therapeutic window where anti-tumor responses could be triggered without detrimental autoimmune toxicity. Additionally, as demonstrated in our own studies, compared to current NK-cell-based therapies, where addition of cytokines, such as IL-2 or interferons, is required to effectively activate the immune system, adoptive transfer of small amounts of TAM-deficient NK cells proved to be sufficient to mediate tumor rejection [29]. Thus, TAM therapies could bypass the need for high toxic doses of adjuvants.

In this framework, and based on the overall literature herein presented, we would like to reinforce the potential therapeutic use of TAM signaling inhibition not only for cancer therapy but, crucially, for cancer immunotherapy. We anxiously await future research that could help translate the exciting experimental observations into the clinics.
